# Retrograde aortic dissection encountered amidst nephrectomy for renal cell carcinoma with IVC thrombus – a case report

**DOI:** 10.1186/s12894-024-01662-x

**Published:** 2024-12-04

**Authors:** Kasi Viswanath Gali, Guruprasad D. Rai, Anupam Choudhary, K. R. Surag, Ganesh S. Kamath, Arun Chawla, Vijay Gunashekar

**Affiliations:** 1 Department of Urology, Kasturba Medical College, Manipal, Manipal Academy of Higher Education, Manipal, Karnataka 576104 India; 2 Department of Cardiothoracic Surgery, Kasturba Medical College, Manipal, Manipal Academy of Higher Education, Manipal, Karnataka 576104 India

**Keywords:** Retrograde Aortic Dissection, Renal Cell Carcinoma, Inferior Vena Cava Thrombus, Cardiopulmonary Bypass, Radical Nephrectomy, Intraoperative Complications

## Abstract

**Background:**

Management of RCC with IVC thrombus can be surgically challenging, particularly when the tumour thrombus extends above the diaphragm. Cardiopulmonary bypass is often employed to aid surgical removal of the tumour in such cases.

**Case presentation:**

We detail an instance of 67-year-old Male patient suffering from RCC with IVC thrombus, with the tumour thrombus extending into the right atrium, who developed on-table retrograde type A aortic dissection amidst the surgical procedure, thereby precluding cardiopulmonary bypass. Transfixation of the renal arterial stump resulted in disappearance of the dissection flap.

**Conclusions:**

Operating surgeons should be mindful of the potential for retrograde aortic dissection during Radical Nephrectomy and its implications intraoperatively.

## Introduction

Renal cell carcinoma (RCC) is a malignancy known for its potential to extend into major blood vessels, posing significant challenges in its management. Approximately 10% of RCC involve extension into the renal vein and inferior vena cava (IVC), while extension into the right atrium is encountered in about only 1% of these patients [1]. The surgical management of RCC with tumor extension into the right atrium requires a multidisciplinary approach and often necessitates the use of cardiopulmonary bypass. Owing to the rarity of this clinical condition, the surgical experience remains limited. Here, we report an unexpected on-table occurrence of retrograde type A aortic dissection (RTAD) during surgery for RCC with an IVC thrombus extending into the right atrium, which subsequently precluded the use of cardiopulmonary bypass.

## Case report

**Fig. 1:**
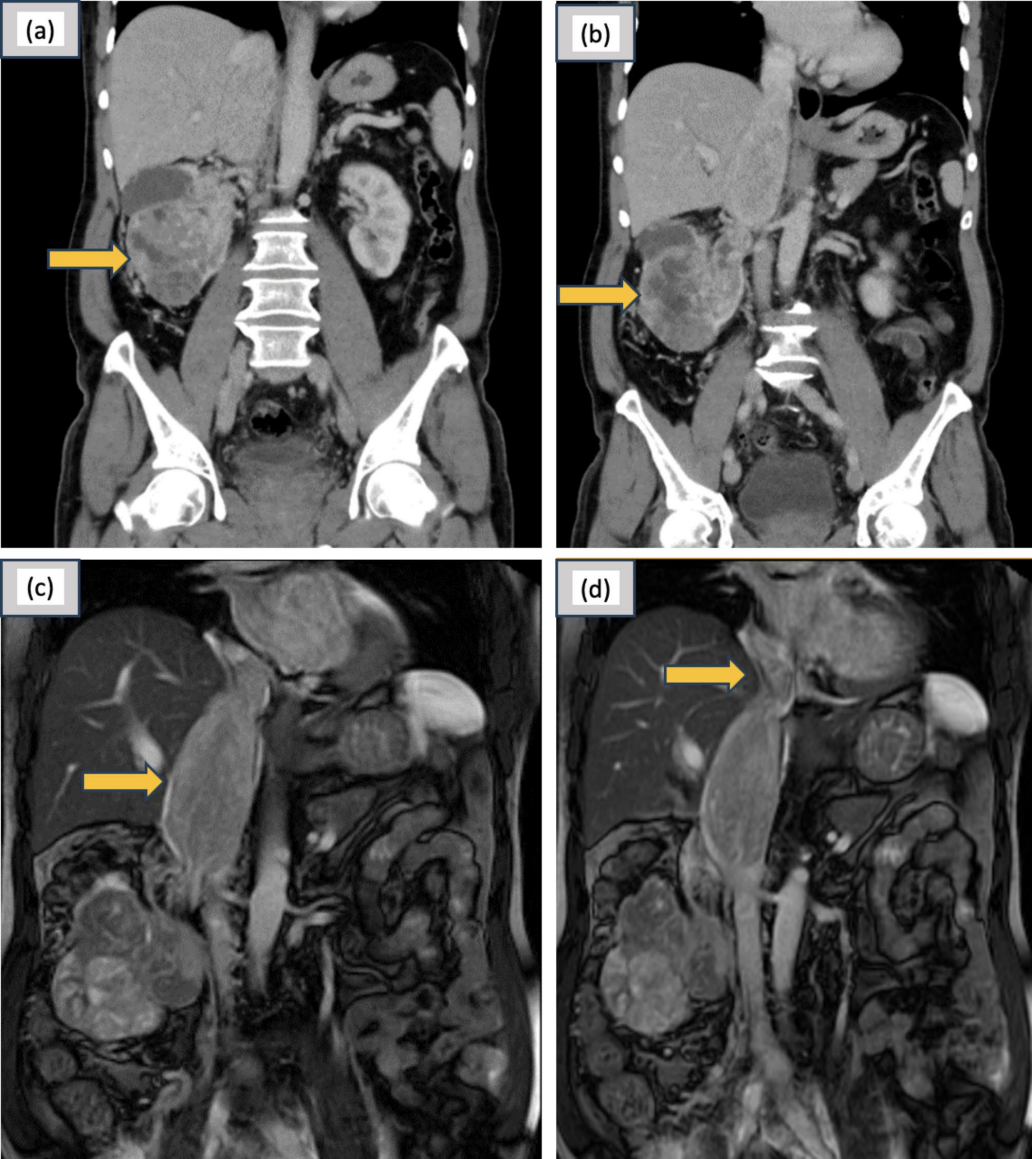
(**a**) & (**b**) CECT images showing the Enhancing Renal Mass; (**c**) & (**d**) MRI images showing the enhancing Tumour Thrombus extending upto the right atrium

A 67-year-old male, hypertensive, was referred to the urology clinic following the incidental discovery of a renal mass during a routine ultrasound abdomen in a general health checkup. This patient, a known smoker for the past 15 years, underwent further evaluation, including a contrast-enhanced computed tomography (CECT) of the abdomen and pelvis (Fig. 1). CECT revealed an exophytic, ball-type mass measuring approximately 11 × 9 × 7 cm involving the cortex and medulla of the mid and lower pole of the right kidney. The lesion displayed necrotic/cystic areas with heterogeneous hyperenhancement during the arterial phase and rapid washout in the nephrogenic phase. Additionally, the right renal vein, the infrahepatic and intrahepatic inferior vena cava (IVC) were dilated, with an enhancing mass lesion within, suggestive of tumor thrombus. Magnetic resonance imaging (MRI) of the abdomen with an inferior venacavogram (Fig. 1) was performed. This revealed an extension of the tumor thrombus measuring 12.7 cm into the suprahepatic IVC and up to the right atrium.

**Fig. 2:**
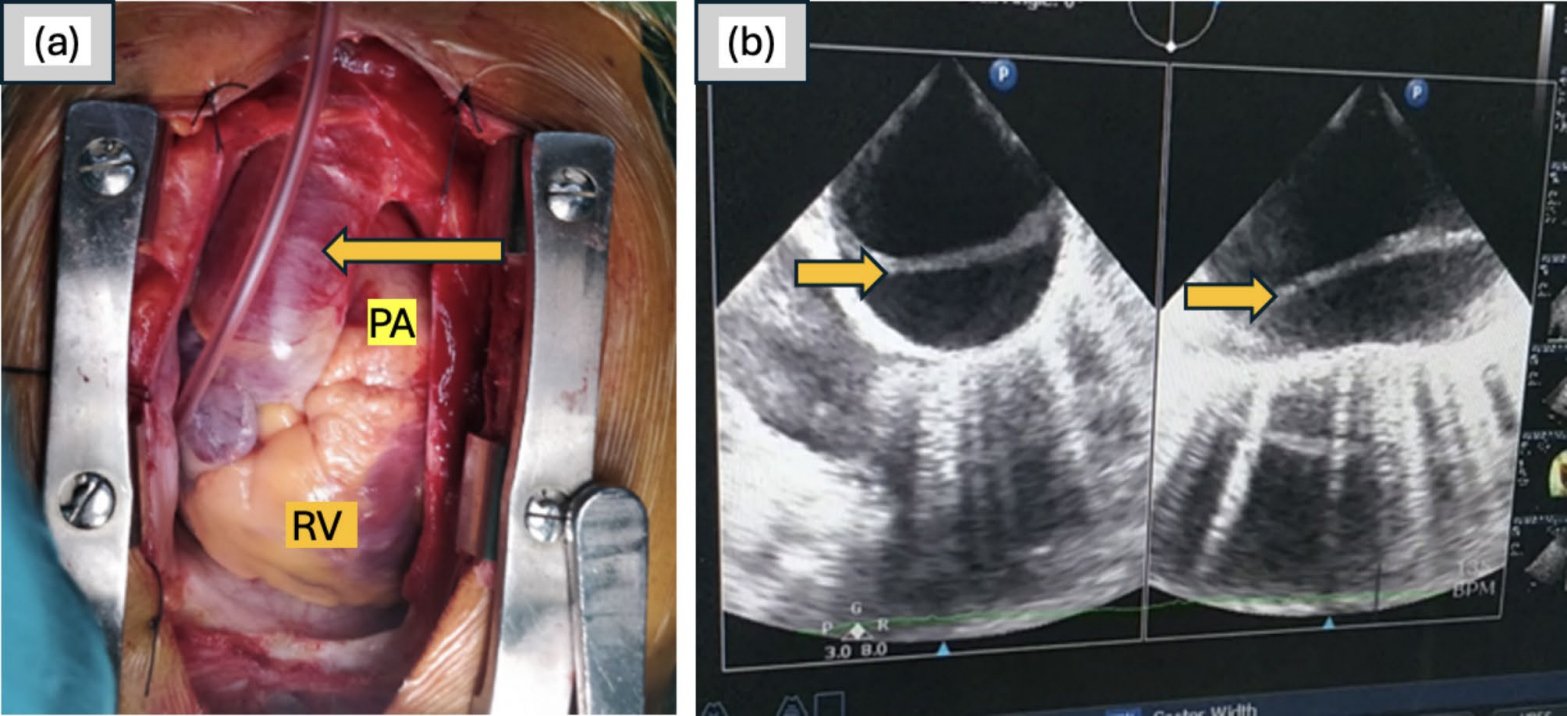
(**a**) Congestion of the aorta; (**b**) Dissection flap noted in the aorta. PA: Pulmonary Artery, RV: Right Ventricle

The patient was scheduled for a Right Radical Nephrectomy with IVC thrombectomy on cardiopulmonary bypass after a comprehensive counselling and informed consent was obtained. The abdominal approach successfully dissected the right renal mass, ligating the artery to expose the vein. A sternotomy preceded aortic cannulation for cardiopulmonary bypass. Unexpectedly, a sudden change in the aorta’s texture and color, coupled with evidence of aortic dissection on transesophageal echo, which was absent earlier (Fig. 2), prompted reevaluation. Severe AR and a flap extending into the aortic annulus were identified. These findings had not been seen on the preoperative MRI and transthoracic echocardiogram, suggesting it was an intraoperative occurrence.

**Fig. 3:**
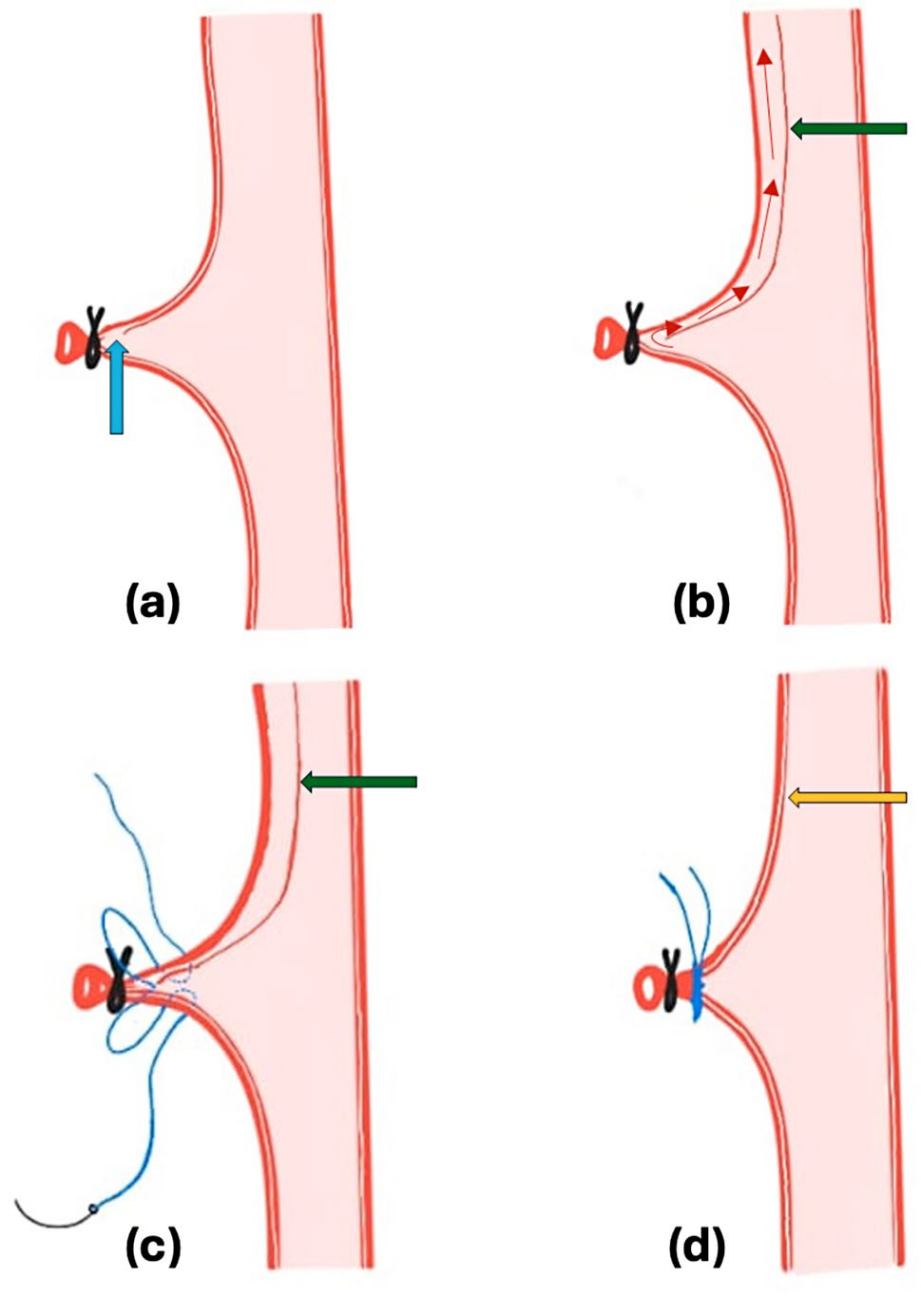
(**a**) Development of a false lumen in the renal arterial stump (blue arrow); (**b**) Appearance of the aortic dissection flap (green arrow); (**c**) Transfixation suture taken on the renal arterial stump using prolene suture; (**d**) Disappearance of the dissection flap (yellow arrow) following the transfixation suture

The cardiovascular team decided against proceeding with cardiopulmonary bypass due to the aortic dissection. A joint intraoperative decision by the urology and CTVS teams was made to first remove the thrombus along with the tumor, followed by repair of the aortic dissection. The intra-pericardial IVC was looped, and a snugger was applied. An incision on the suprarenal IVC, along with controlled traction, facilitated thrombus extraction. Once the intra-pericardial IVC was clear, the snugger was tightened. Complete removal of the renal mass and IVC thrombus was accomplished. Following extraction of the specimen, a small bleeder from the right renal artery stump prompted reinforcement with a transfixation suture using Prolene (Fig. 3). Transfixing the renal artery, caused near-complete resolution of the dissection flap, indicating a retrograde nature of aortic dissection (RTAD). A postoperative transesophageal echocardiogram revealed a normal aorta with no flap, and aortic regurgitation was trivial. The postoperative course of the patient was uneventful and was discharged on POD 6. Histopathological examination revealed clear cell renal cell carcinoma and the one-month follow-up demonstrated a positive recovery trajectory with no reported complications.

## Discussion

The management of RCC with tumour thrombus, extending upto right atrium requires a multidisciplinary approach in its management, considering the involvement of vital structures and potential surgical morbidity [2]. Imaging modalities such as CECT, MRI, and Transesophageal/transthoracic echocardiogram are an integral part of evaluation. While considering surgical removal in this scenario, careful preoperative planning is crucial. Complete cardiopulmonary bypass may become essential in cases where the tumor extends into the supradiaphragmatic intrapericardial inferior vena cava (IVC) or infiltrates the right atrium and extends further, requiring atriotomy for the management of level IV tumor thrombi [3]. Intraoperative use of Transesophageal echocardiography (TEE) is the standard of care during surgeries for RCC with IVC thrombus at our institution. It allows for real-time monitoring of the tumor thrombus throughout the procedure, to detect any embolization of tumour thrombus during manipulation and also helps confirm complete thrombus removal. Collaborative efforts between urologists, cardiovascular specialists and a dedicated Anaesthetic team are imperative in devising comprehensive treatment strategies tailored to individual cases.

The actual occurrence rate of RTAD remains undetermined, but it is increasingly recognized, with an estimated overall frequency ranging from 1 to 4%. Modalities for diagnosing RTAD include transesophageal echocardiography, CT angiography, and MRI. Aortic specialists need to be vigilant as RTAD can manifest either acutely or with a delayed onset, potentially occurring during the indexed TEVAR procedure or in the postoperative period [4]. In cases of RTAD, the primary entry tear typically arises distal to the left subclavian artery (LSA), leading to retrograde dissection into the ascending aorta. RTAD can be spontaneous or iatrogenic, linked to both open and endovascular aortic surgeries, particularly when the proximal aorta exceeds a dilation of 40 mm [5]. To our knowledge, this is the first reported case of RTAD occurring during radical nephrectomy. Although urologists rarely encounter this complication during nephrectomy, acute RTAD is a feared occurrence that may go unnoticed on numerous occasions. Utilization of TEE in our case along with the visible color change helped identify this rare yet significant complication.

The management of spontaneous RTAD currently lacks standardized protocols, with documented success in treatment using optimized medical therapy, open surgery, and/or endovascular repair [6]. In our case, we narrowly averted a critical situation as the false lumen was obliterated upon transfixing the renal artery, indicating that the origin of the dissection was probably from the renal artery and had progressed to the ascending aorta (Fig. 3). Conversely, a fatal outcome could have ensued if the patent false lumen in the ascending aorta had not closed, necessitating prompt surgical intervention to address complications such as pericardial effusion, aortic regurgitation, or malperfusion. As this was our first encounter with such a complication, we cannot definitively suggest preventive measures; however, using a transfixation suture for ligation might help. The operating surgeons should thereby be mindful of the potential for retrograde aortic dissection and its implications during radical nephrectomy and IVC Thrombectomy, as seen in our case. The long-term prognosis following such an event requires further study, necessitating a stringent follow-up schedule and multidisciplinary assessment. Based on our case, the prognosis appears favorable provided resolution of the dissection flap is achieved, with no concerning sequelae observed so far, although extended follow-up remains necessary.

## Conclusions

Urologists should be aware of the potential for retrograde aortic dissection during Radical Nephrectomy and its clinical implications, which are best managed through a multidisciplinary approach.

## Data Availability

The datasets used and/or analyzed during the current study are available from the corresponding author on reasonable request.

## Abbreviations

RCC: Renal Cell Carcinoma
IVC: Inferior Vena Cava
RTAD: Retrograde Type A Dissection
CECT: Contrast Enhanced Computed Tomography
MRI: Magnetic Resonance Imaging
FDG PET: Fluorodeoxyglucose-Positron Emission Tomography
TEVAR: Thoracic endovascular aortic repair
